# Supplementary figures and images for: A RID-like putative cytosine methyltransferase homologue controls sexual development in the fungus Podospora anserina
Source: PLoS Genet. 2019 Aug 14;15(8):e1008086. doi: 10.1371/journal.pgen.1008086 (PMC6709928; doi:10.1371/journal.pgen.1008086)

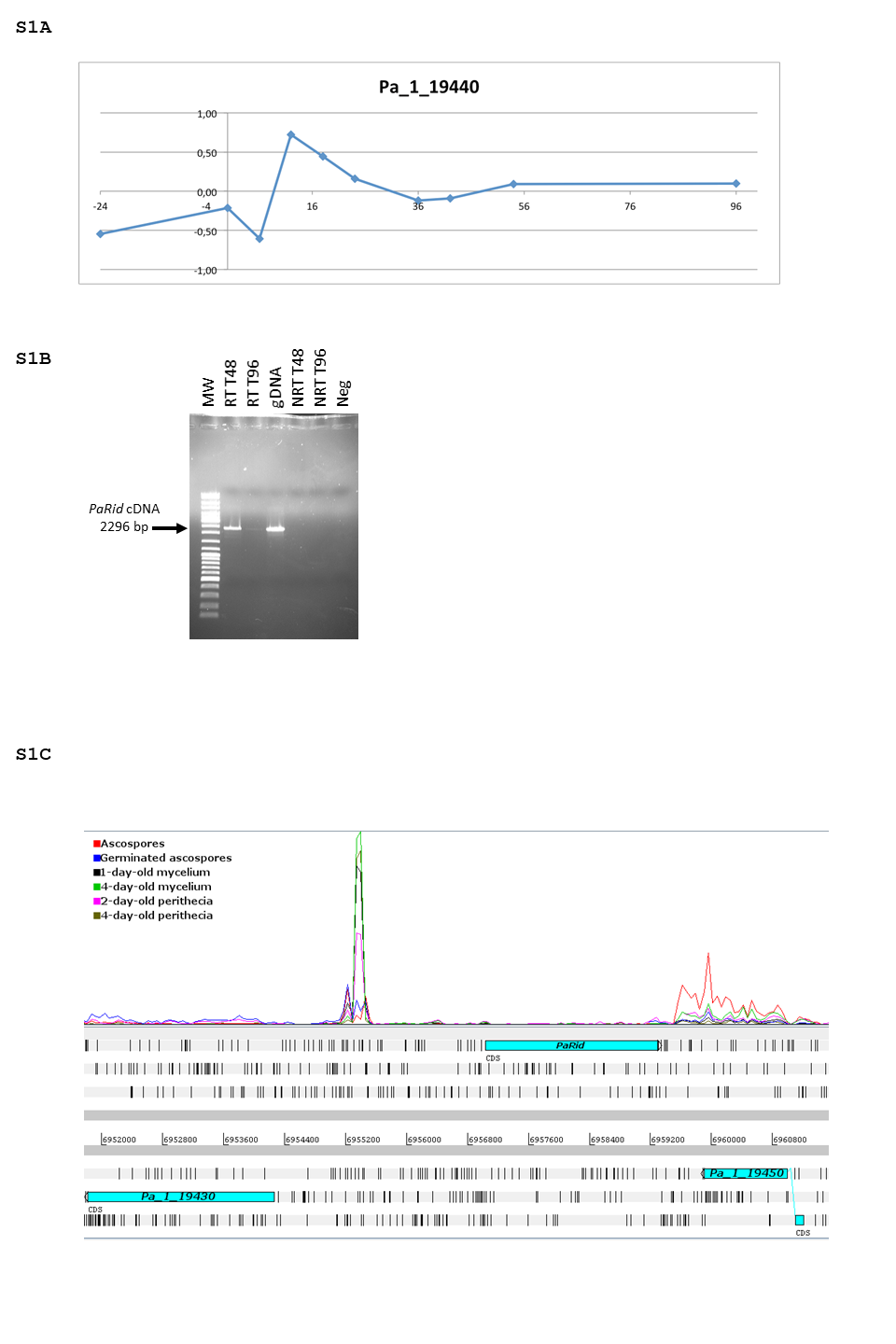

Supplement: S1 Fig — (A) Average expression profiles (y-axis) of PaRid (Pa_1_19440) during sexual development (x-axis, hours). (B) Amplification of PaRid transcripts (2296 bp) by RT-PCR. MW: GeneRuler DNA Ladder Mix (Thermo Fisher Scientific), RT T48, RT T96: RT-PCR performed on RNA extracted from 2 days or 4 days post fertilization developing perithecia, gDNA: genomic DNA, NRT: PCR performed on RNA extracted from 2 days or 4 days post fertilization developing perithecia, Neg: No RNA. See (Materials and methods section for details). (C) Coverage of RNA-seq mapped reads at the PaRid locus [37]. RNA-seq experiments were performed on RNAs extracted from non-germinated ascospores (Ascospores), eight hours germinating ascospores (Germinated ascospores), 1-day- or 4-day-old mycelia, 2 days or 4 days post fertilization developing perithecia. (TIF) [file pgen.1008086.s001.tif]

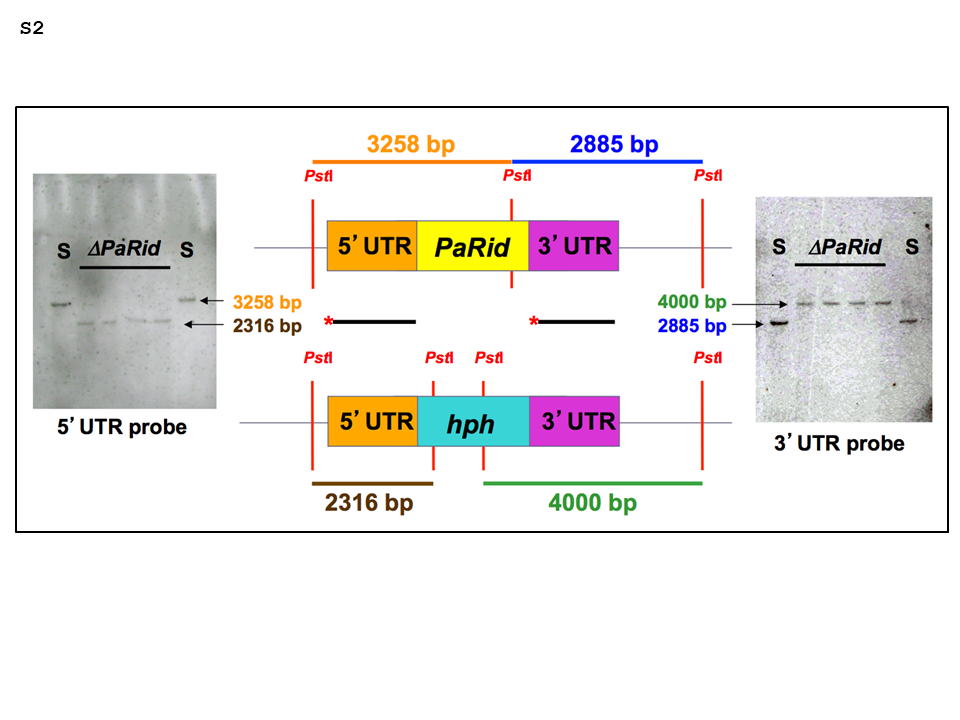

Supplement: S2 Fig — Schematic representations of the endogenous and disrupted loci are given in. Replacement by homologous recombination of the wild type PaRid allele by the disrupted ΔPaRid allele results in the substitution of a 3.2 kb PstI fragment by a 2.3 kb PstI fragment as revealed by hybridization of the 5’UTR digoxygenin-labeled probe (S2A) and in the substitution of a 2.8 kb PstI fragment by a 4.0 kb PstI fragment as revealed by hybridization of the 3’UTR digoxygenin-labeled probe (S2A). (TIF) [file pgen.1008086.s002.tif]

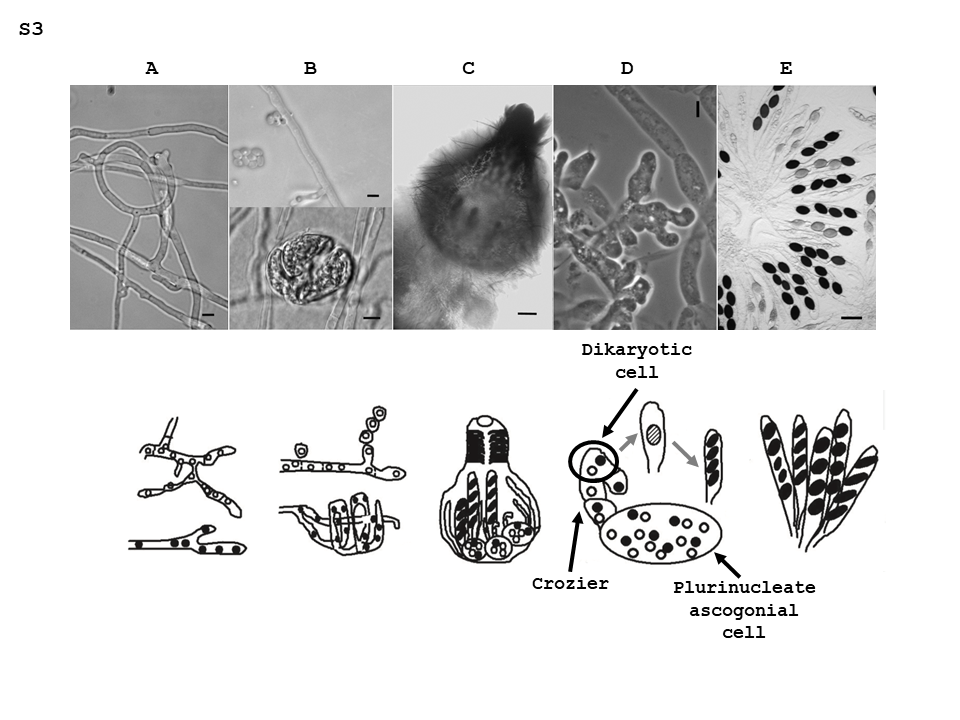

Supplement: S3 Fig — Major steps of P. anserina’s life cycle as shown by a schematic representation (upper panel) and the corresponding light microphotographs (lower panel). P. anserina’s life cycle begins with the germination of an ascospore (A) that gives rise to a haploid mycelium (B). After three days of growth, both male gametes (spermatia, B, top) and female gametes (ascogonia, B, bottom) are formed. Because most of the ascospores carry two different and sexually compatible nuclei (mat+ and mat- mating types) P. anserina strains are self-fertile (pseudo-homothallism). Before fertilization occurs, ascogonia can mature into protoperithecia by recruiting protective maternal hyphae to shelter the ascogonial cell. A pheromone/receptor signaling system allows the ascogonia to recognize and fuse with spermatia of compatible mating type (heterothallism). Fertilization initiates the development of the fruiting body (perithecia, C) in which the dikaryotic mat+/mat- fertilized ascogonium forms. Further development leads to a three-celled hook-shaped structure called the crozier (D). The two parental nuclei in the middle cell of the crozier fuse (karyogamy, schematic representation D) to form a diploid nucleus, which then immediately undergoes meiosis. The four resulting haploid nuclei undergo mitosis. In most cases, ascospores are formed around 2 non-sister nuclei within the developing ascus. On rare occasions, two ascospores are formed around only one haploid nucleus each, leading to a five-ascospore ascus (E, photograph). Scale bar: 10 μm in (A-D); 200 μm in (E). (TIF) [file pgen.1008086.s003.tif]

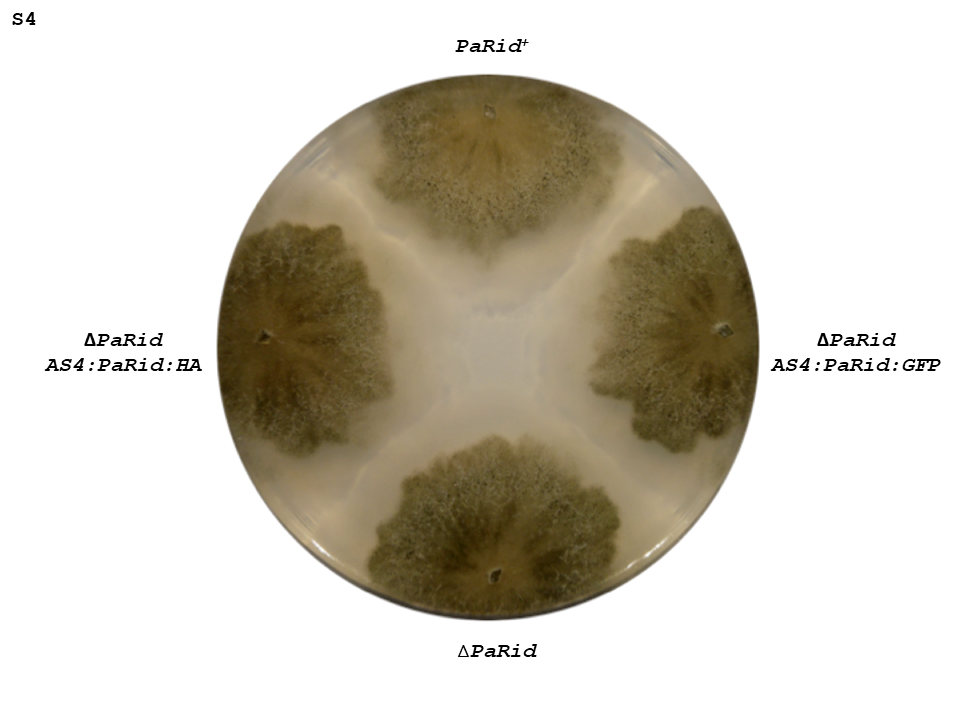

Supplement: S4 Fig — Strains were grown on M2 minimal medium for 6 days at 27°C. S: Wild-type strain. See Materials and Methods section for details on the complemented ΔPaRid:AS4:PaRid:HA strain. (TIF) [file pgen.1008086.s004.tif]

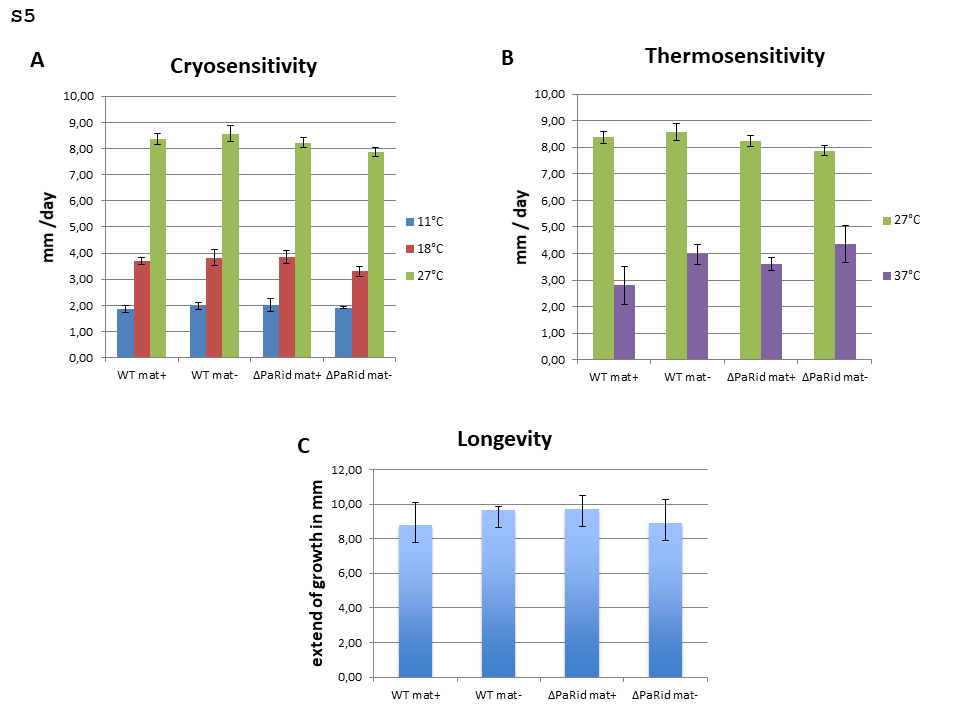

Supplement: S5 Fig — (A) Cryosensitivity assay: growth rate at two sub-optimal temperatures compared with optimal temperature. (B) Thermosensitivity assay: growth rate at 37°C compared with optimal temperature. (C) Longevity assay. For each genotype, growth was assayed on 3 independent cultures after 4 days of growth at the indicated temperatures. Each experiment was performed three times. For each genotype, longevity was measured on three independent cultures, issued from three individual ascospores, as described in [42]. (TIF) [file pgen.1008086.s005.tif]

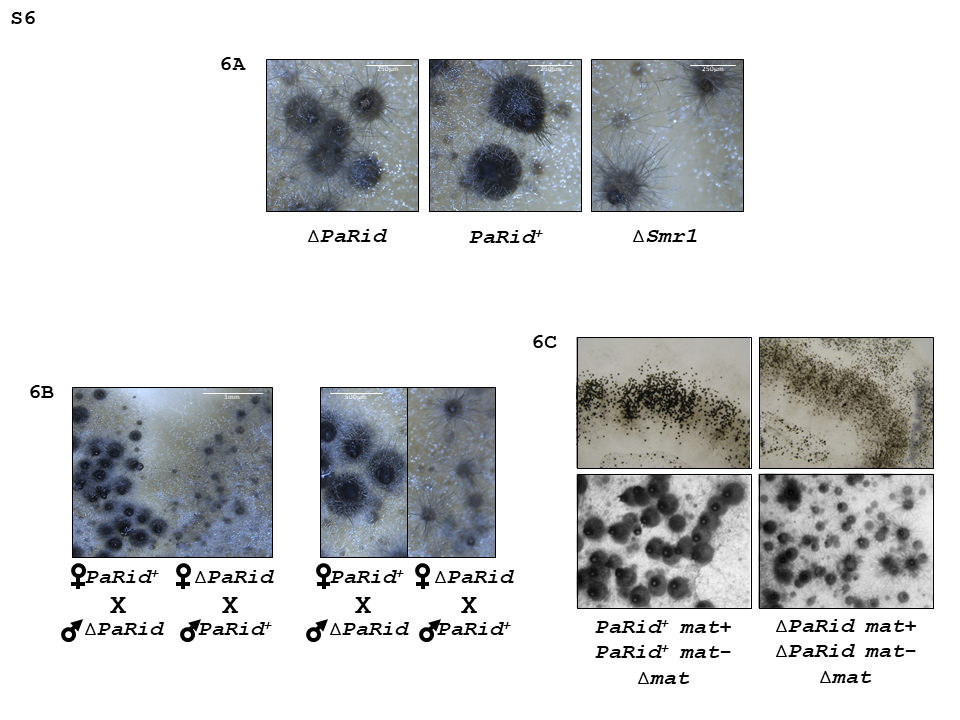

Supplement: S6 Fig — (A) Morphological comparison of perithecia obtained from wild-type crosses (PaRid), ΔPaRid crosses (ΔPaRid) and ΔSmr1 crosses (ΔSmr1). Size and morphology of the ΔPaRid and ΔSmr1 perithecia are alike. Scale bar: 250 μm. (B) Perithecia obtained in the indicated trikaryons on M2 medium after 5 days at 27°C. The Δmat; PaRid+ mat-; PaRid+ mat+ trikaryons form typical fully developed perithecia (left panels). By contrast, only blocked micro-perithecia are formed by the Δmat; ΔPaRid mat-; ΔPaRid mat+ trikaryons (right panels). (C) Heterozygous orientated crosses PaRid+ mat- × ΔPaRid mat+ after 5 days at 27°C. When the wild-type PaRid+ allele is present in the female gametes genome and the mutant ΔPaRid allele is present in the male gamete genome fully developed perithecia are formed, conversely when the mutant ΔPaRid allele is present in the female gametes and the wild-type PaRid+ allele is present in the male gamete, only blocked micro-perithecia are formed. Left panel, scale bar: 1 mm, right panel, scale bar: 500 μm. (TIF) [file pgen.1008086.s006.tif]

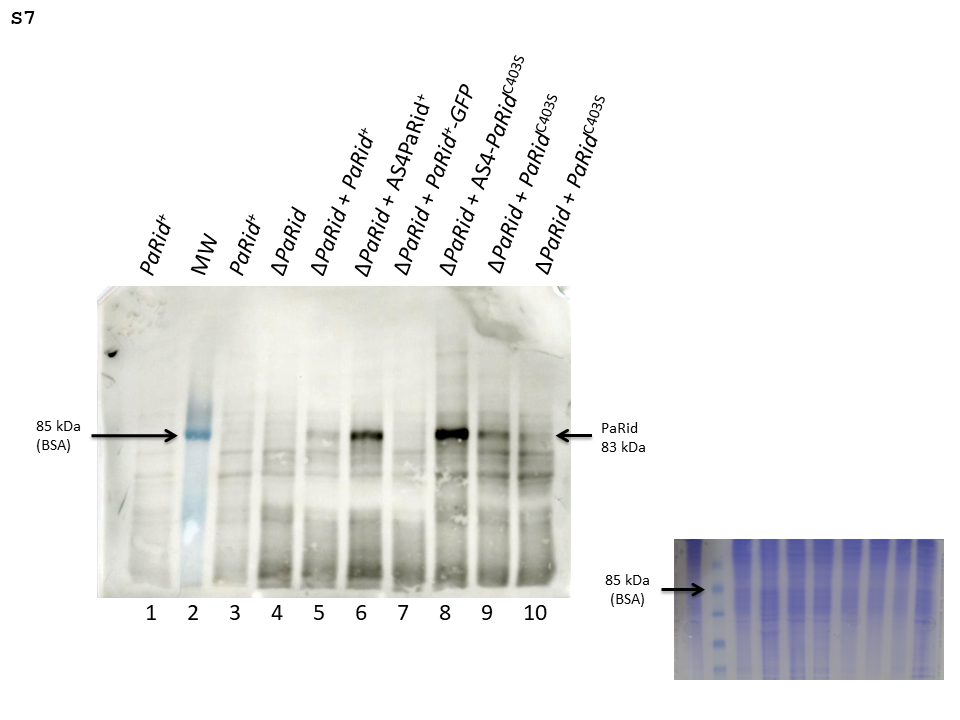

Supplement: S7 Fig — Western-blot was performed as described in Material and Methods section and probed with an anti-HA antibody that specifically detects the HA-tagged proteins. The PaRid protein (83 kDa) was barely detectable when the expression of the ectopic alleles was driven from its native promoter (ΔPaRid:PaRid, ΔPaRid:PaRidC403S). However, it was readily produced when the expression of the ectopic alleles was under the control of a strong and constitutive promoter (ΔPaRid:AS4-PaRid, ΔPaRid:AS4-PaRidC403S). Complementation of the fertility defect was obtained by insertion of the wild type PaRid-HA allele or by insertion of the AS4-PaRid-HA only. Negative controls (No PaRid-HA tagged): PaRid+ wild-type strains mat+ (lane 1) & mat- (lane 3), ΔPaRid mutant strain (lane 4), ΔPaRid:PaRid+-GFP strain (no HA tag, GFP only, complemented strain, lane 7). ΔPaRid:PaRid+ = ΔPaRid mutant strain harboring an ectopic wild type PaRid+-HA allele (complemented strain, lane 5), ΔPaRid:AS4-PaRid = ΔPaRid mutant strain harboring an ectopic AS4-PaRid+-HA allele (complemented strain, lane 6), ΔPaRid:AS4-PaRidC403S = ΔPaRid mutant strain harboring an ectopic AS4-PaRidC403S-HA allele (non-complemented strain, lane 8). ΔPaRid:PaRidC403S = ΔPaRid mutant strain harboring an ectopic catalytically dead PaRidC403S-HA allele (two non-complemented strains, lane 9 & 10), MW: Prestained Protein Molecular Weight Marker (Thermo Scientific, lane 2). See the material and method section for details on the alleles construction and features. (TIF) [file pgen.1008086.s007.tif]

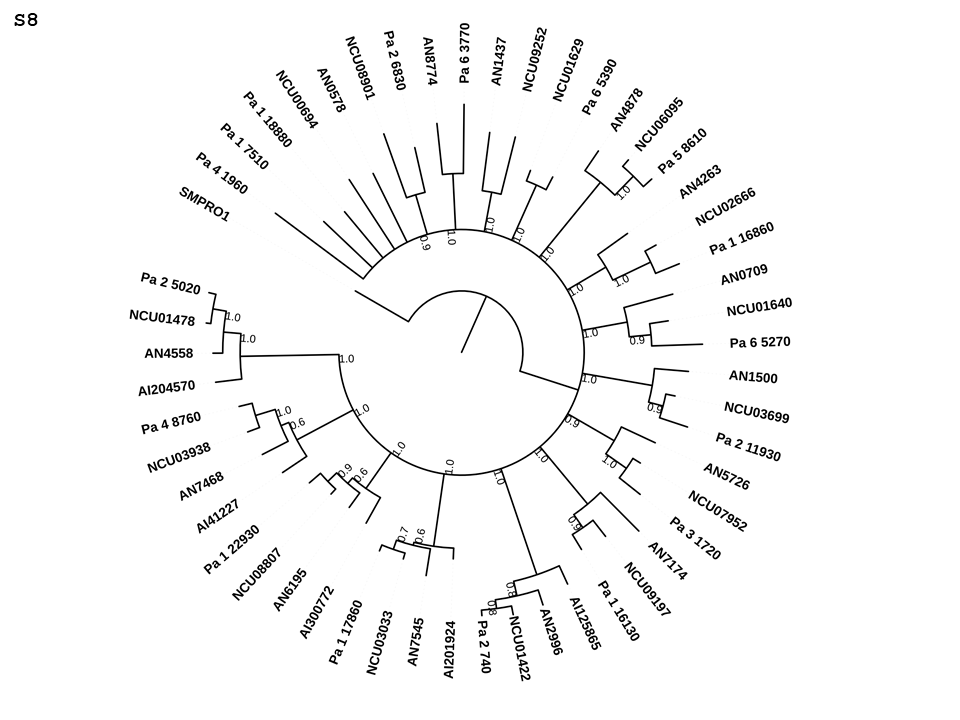

Supplement: S8 Fig — If four out of 17 have orthologs in A. nidulans, A. immersus and N. crassa (Pa_1_17860, Pa_1_22930, Pa_2_740 and Pa_2_5020), Pa_4_1960 is the only TF of the set showing no orthologs into the genomes of these three species. Although Pa_1_18880 and Pa_7510 display orthologs either in N. crassa or in N. crassa and A. nidulans, their phylogenetic positions were ambiguous, suggesting some species specialization. See S6 Table for protein names. Only bootstrap values > 0.5 are indicated on the corresponding branches. (TIF) [file pgen.1008086.s008.tif]
