# Supplementary material for: A RID-like putative cytosine methyltransferase homologue controls sexual development in the fungus Podospora anserina
Source: PLoS Genet. 2019 Aug 14;15(8):e1008086. doi: 10.1371/journal.pgen.1008086 (PMC6709928; doi:10.1371/journal.pgen.1008086)
Supplement: S1 Table — (DOCX) [file pgen.1008086.s009.docx]

**S1 Table. Primers used in this study.**

| Primer Names | Primer sequences |
| --- | --- |
|  |  |
| ΔRID2F | CATTGGTCTTCCCAGTGACGTAACCAAC |
| ΔRID2R | cttaccgctgttgagatccagttcgatGAGAAGGAGCAAGAAGGATAAGGGCAACC |
| ΔRID3F | GGTTGCCCTTATCCTTCTTGCTCCTTCTCatcgaactggatctcaacagcggtaag |
| ΔRID3R | GCTTCATAAGATCGCCAATAACATGCCACggttcagggcagggtcgttaaatag |
| ΔRID4F | ctatttaacgaccctgccctgaaccGTGGCATGTTATTGGCGATCTTATGAAGC |
| ΔRID4R | GCCAAATTCTGAACAGGGGTAGCTTTG |
|  |  |
| RIDmut1 | TCCCCTCCGT***C***TCAGTTCTGGTCCCCGGC |
| RIDmut2 | CAGAACTGA***G***ACGGAGGGGAGAGGTGGAGG |
| PaRIDHAFXbaI | A*TCTAGA*TCAAAATCACTGACCACTCAGC |
| PaRIDHAREcoRI | A*GAATTC*CGTCTCCCAATCAATAACC |
| AS4PaRIDmutF | gtgatcgcaaaaccgtcaggaattACAAAAAAATGGCCGAAATTTACAACCCG |
| PaRIDmutAS4R | cgggttgtaaatttcggccatttttttgtAATTCCTGACGGTTTTGCGATCAC |
| PaRIDAS4XbaI | A*TCTAGA*CGTCTCCCAATCAATAACC |
| RIDGFPFEcoRI | A*GAATTC*ATGGTGAGCAAGGGCGAGGAGC |
| RIDGFPRClaI | A*ATCGAT*GGCGCTTGACTTGTACAGCTCGTCC |
|  |  |
|  |  |
| NcRIDHAFNotI | A*GCGGCCGC*TGGAGGTATCCATTGACTTTCC |
| NcRIDHARBamHI | A*GGATCC*TAGTCTTGAGTCGTCGAAAAGCTCC |
| dmtAFXbaI | A*TCTAGA*ATGAGACAGTCAGCCTATATTTACCTAAC |
| dmtARBamHI | A*GGATCC*TTGGTTTTCGTGCAATTCAC |
| AiRIDAS4XbaI | A*TCTAGA*ACACCATGTCGGAAAGAAGATACG |
| AiRIDHAREcoRI | A*GAATTC*CTCCATCCTCTCGAAATCCTTCTCC |
|  |  |
|  |  |
| PaRIDF1 | CGGTTGCCGACTTCTGTT |
| PaRIDF2 | GGACATTCTCCAATACTGGACA |
| PaRIDF3 | TTCACTGATCTGGAATATGCTG |
| PaRID1R | TCGGGTGTTTTGGAGTGG |
|  |  |
| NcRIDF2 | AACATCTTGATGACATTCAG |
| NcRIDF3 | ATCGATATTACAGAAGACG |
| NcRIDF4 | ATGGTCATTTATTAGAGTGG |
| NcRIDF4bis | TGTCGAACAAACGTTCGGC |
| NcRIDF5 | TCGAATGCTTAGATAAACGGG |
| NcRIDF6 | ACAAGGACGGGTTCAAAGGCC |
| NcRIDF7 | TCTTGAAGGAGTTGGGCGG |
|  |  |
| AiRIDF3 | TGTCGAAGCGCTTCGTCCTACGGG |
|  |  |
| 5s-PaRid | gttgttactcacacaaaaaaatgg |
| 3s-PaRid | ccactctcctgccaaaagtcaatc |
