# Supplementary material for: A RID-like putative cytosine methyltransferase homologue controls sexual development in the fungus Podospora anserina
Source: PLoS Genet. 2019 Aug 14;15(8):e1008086. doi: 10.1371/journal.pgen.1008086 (PMC6709928; doi:10.1371/journal.pgen.1008086)
Supplement: S2 Table — (DOCX) [file pgen.1008086.s010.docx]

**S2 Table. Vegetative phenotypes analyses.**

|  | *PaRid^+^* | *ΔPaRid* |
| --- | --- | --- |
| Growth rate on M2 |  |  |
| Germination | 100 % | 100 % |
| Senescence |  |  |
| Cell fusion | Efficient | Efficient |
| Vegetative Incompatibility S vs s | Cell death | Cell death |
| Neutral osmo-sensitivity  (saccharose 200g/l) | 100 % | 100 % |
| Neutral osmo-sensitivity  (sorbitol 200g/l) | 100 % | 100 % |
| Ionic sensitivity  (KCl 0.5M) | 100 % | 100 % |
| Ionic sensitivity  (NaCl 0.5M) | 100 % | 100 % |
| 10^-4^ M TBY sensitivity | 100 % | 100 % |
| 5. 10^-5^ M Menadione sensitivity | 100 % | 100 % |
| 50 μg/ml calcofluor sensitivity | 100 % | 100 % |
| 5mM caffeine sensitivity | 100 % | 100 % |

For each condition, 3 independent experiments were performed.
