# Supplementary material for: A RID-like putative cytosine methyltransferase homologue controls sexual development in the fungus Podospora anserina
Source: PLoS Genet. 2019 Aug 14;15(8):e1008086. doi: 10.1371/journal.pgen.1008086 (PMC6709928; doi:10.1371/journal.pgen.1008086)
Supplement: S3 Table — (DOCX) [file pgen.1008086.s011.docx]

**S3 Table. Sexual reproduction phenotypic analyses.**

|  | *PaRid^+^ mat+ X PaRid^+^ mat-* | *ΔPaRid mat+ X ΔPaRid mat-* |
| --- | --- | --- |
| Spermatia | 1.55*10^6^ +/- 3.77*10^5^ | 1.4*10^6^ +/- 3.28*10^5^ |
| Perithecia | [WT]  4477 ± 456 | [**micro**]  4333 ± 368 |
| Ascospores production  (96H post-fertilization) | WT production | **None** |

For each condition, 5 independent experiments were performed.
