# Supplementary material for: A RID-like putative cytosine methyltransferase homologue controls sexual development in the fungus Podospora anserina
Source: PLoS Genet. 2019 Aug 14;15(8):e1008086. doi: 10.1371/journal.pgen.1008086 (PMC6709928; doi:10.1371/journal.pgen.1008086)
Supplement: S4 Table — (DOCX) [file pgen.1008086.s012.docx]

**S4 Table. Grafting experiments**

| Recipient strain | *PaRid^+^* | | *ΔPaRid* | |
| --- | --- | --- | --- | --- |
| Grafted perithecia | *PaRid^+^* | *ΔPaRid* | *PaRid^+^* | *ΔPaRid* |
| Perithecium phenotype | [WT] | [**micro**] | [WT] | [**micro**] |
| Ascospore production | [WT] | **None** | [WT] | **None** |

Grafted perithecia per experiment N=9, 3 independent experiments
