# Supplementary material for: A RID-like putative cytosine methyltransferase homologue controls sexual development in the fungus Podospora anserina
Source: PLoS Genet. 2019 Aug 14;15(8):e1008086. doi: 10.1371/journal.pgen.1008086 (PMC6709928; doi:10.1371/journal.pgen.1008086)
Supplement: S7 Table — (DOCX) [file pgen.1008086.s015.docx]

**S7 Table. Strains used in this study**

| Alleles | Promoter | Expressed protein | Number of independent tested transformants |
| --- | --- | --- | --- |
| *PaRid* *mat+* | Native PaRid | Wild-type PaRid | 2 |
| *PaRid* *mat-* | Native PaRid | Wild-type PaRid | 2 |
| Δ*PaRid* *mat+* | None | None | 2 |
| Δ*PaRid* *mat-* | None | None | 2 |
| Δ*mat* | Native PaRid | Wild-type PaRid | 1 |
| *PaRid-HA* | Native PaRid | Wild-type PaRid HA tagged | 109 |
| *PaRid-GFP-HA* | Native PaRid | Wild-type PaRid HA and GFP tagged | 63 |
| *AS4-PaRid-HA* | Highly and constitutively active *AS4* | Wild-type PaRid HA tagged | 78 |
| *AS4-PaRid-GFP-HA* | Highly and constitutively active *AS4* | Wild-type PaRid HA and GFP tagged | 95 |
| *PaRid*^C403S^*-HA* | Native PaRid | Point-mutated catalytically dead HA tagged | 89 |
| *AS4-PaRid*^C403S^*-HA* | Highly and constitutively active *AS4* | Point-mutated catalytically dead HA tagged | 55 |
